# Melatonin Suppresses the Kainate Receptor-Mediated Excitation on Gonadotropin-Releasing Hormone Neurons in Female and Male Prepubertal Mice

**DOI:** 10.3390/ijms21175991

**Published:** 2020-08-20

**Authors:** Santosh Rijal, Dong Hyu Cho, Seon-Ah Park, Seon Hui Jang, István M. Ábrahám, Seong Kyu Han

**Affiliations:** 1 Department of Oral Physiology, School of Dentistry & Institute of Oral Bioscience, Jeonbuk National University, Jeonju 54896, Korea; santoshrijal047@gmail.com (S.R.); sunnyjang@jbnu.ac.kr (S.H.J.); 2 Department of Obstetrics and Gynecology, Jeonbuk National University Medical School, Institute of Clinical Medicine of Jeonbuk National University-Biomedical Research Institute and Institute for Medical Sciences, Jeonbuk National University Hospital, Jeonju 54907, Korea; obgyn2001@jbnu.ac.kr; 3 Non-Clinical Evaluation Center, Biomedical Research Institute, 20 Geonji-ro, Deokjin-gu, Jeonju-si, Jeollabuk-do 54907, Korea; topaz6183@jbnu.ac.kr; 4 PTE-NAP Molecular Neuroendocrinology Research Group, Institute of Physiology, Medical School, Centre for Neuroscience, Szentágothai Research Center, University of Pécs, 7624 Pécs, Hungary; istvan.abraham@aok.pte.hu

**Keywords:** gonadotropin-releasing hormone neuron, kainate, melatonin, G-protein-coupled receptors, patch clamp

## Abstract

Melatonin, a pineal gland secretion, is an amphiphilic neurohormone involved in the biological and physiologic regulation of bodily functions. Numerous studies have shown the effects of melatonin on the release of gonadotropins and their actions at one or several levels of the hypothalamic–pituitary–gonadal axis. However, direct melatonin action on gonadotropin-releasing hormone (GnRH) neurons and its mechanism of action remain unclear. Here, plasma melatonin levels were measured and the effect of melatonin on GnRH neurons was assessed using brain slice patch clamp techniques. The plasma melatonin levels in prepubertal mice were higher than those in the adults. Melatonin itself did not change the firing activity of GnRH neurons. Interestingly, the kainate receptor-mediated responses but not the α-amino-3-hydroxy-5-methyl-4-isoxazolepropionic acid (AMPA)- and N-methyl-D-aspartic acid (NMDA)-induced responses were suppressed by melatonin in both the voltage clamp and current clamp modes. The inhibitory effects of the kainate-induced response by melatonin tended to increase with higher melatonin concentrations and persisted in the presence of tetrodotoxin, a voltage-sensitive Na^+^ channel blocker, or luzindole, a non-selective melatonin receptor antagonist. However, the response was completely abolished by pretreatment with pertussis toxin. These results suggest that melatonin can regulate GnRH neuronal activities in prepubertal mice by partially suppressing the excitatory signaling mediated by kainate receptors through pertussis toxin-sensitive G-protein-coupled receptors.

## 1. Introduction

Gonadotropin-releasing hormone (GnRH) neurons are the central regulators of reproduction and are involved in the pulsatile release of gonadotropins required for puberty and fertility [1]. GnRH neurons express glutamate receptor subunits required for α-amino-3-hydroxy-5-methyl-4-isoxazolepropionic acid (AMPA), N-methyl-D-aspartic acid (NMDA), and kainate signaling [2]. Glutamate, the principal excitatory neurotransmitter in the central nervous system (CNS), regulates GnRH neurons and plays a pivotal role in the initiation of puberty by stimulating GnRH secretion directly or via regulatory neuronal subsets [3]. In addition, glutamate excites GnRH neurons via glutamatergic innervation and stimulates GnRH release from the adult hypothalamus via the activation of kainate and NMDA receptors [4].

Melatonin (N-acetyl-5-methoxytryptamine) is a neurohormone secreted by the pineal gland and is involved in the biological and physiological regulation of bodily functions such as the sleep–wake cycle, pubertal development, seasonal adaptation [5,6], and seasonal reproduction [7]. Pineal melatonin is released into the cerebrospinal fluid (CSF) followed by perfusion of the surface of the CNS [8,9] and alters the function of neurons via G-protein-coupled melatonin receptors such as MT_1_ and MT_2_, which are distributed throughout the CNS [10]. Melatonin has been reported to be an inhibitory factor because melatonin secretion showed a suppressive influence on the hypothalamic secretion of GnRH and the hypothalamus–pituitary–gonadal (HPG) axis [11]. Moreover, a number of studies demonstrated the effect of melatonin on fertility at various levels, including the hypothalamic GnRH neurons, the pituitary gland, gonads, and reproductive tissues [12]. For example, melatonin downregulated the GnRH gene in an immortalized GT1-7 cell line [13] and suppressed GnRH release through melatonin receptors [14]. In addition, melatonin injections to hamsters demonstrated an inhibition of the hypothalamic pulsatile release of GnRH [15] and suppressed fertility via the pituitary-gonadal axis [16]. Likewise, a daily chronic injection of melatonin severely depressed gonadal function in both male and female hamsters [17].

Several studies have shown the high density of melatonin-binding sites present on the pars tuberalis (PT) [18] and a low density of melatonin-binding sites throughout the hypothalamus [19]. Interestingly, the action of melatonin on fertility involves the binding of melatonin to the hypothalamus but not to the PT [19,20]. For example, implanting melatonin-containing pellets in the anterior hypothalamic area and medial preoptic area resulted in the regression of the gonads [21]. Melatonin’s action on the hypothalamus is well documented [19,20,21]. However, its action on hypothalamic GnRH neurons is less reported. Gundogan and colleagues suggested that GnRH neurons were not directly regulated by melatonin in sheep but through a complex neural circuit of interneurons that included excitatory aminoacidergic, dopaminergic, and serotoninergic neurons [22].

Exogenous melatonin is reported to alter the timing of puberty in various animals [23,24,25]. In prepubertal rodents, exogenous melatonin has pronounced anti-gonadal effects [23] and affects pubertal development [24]. Moreover, exogenous melatonin infusion exerted a rapid gonadal response in prepubertal mice compared to adults [26]. Although a number of studies have indicated that melatonin controls fertility at various levels of the HPG axis, there are few reports of the direct action of melatonin on GnRH neurons. In this study, we measured the plasma melatonin levels in C57BL/6 mice and examined the melatonin effect on the ionotropic glutamate receptor-mediated actions of GnRH neurons in prepubertal mice by patch clamp electrophysiology.

## 2. Results

### 2.1. Plasma Melatonin Levels in GnRH-Green Fluorescent Protein-Tagged (GnRH-GFP) Mice

**Figure 1:**
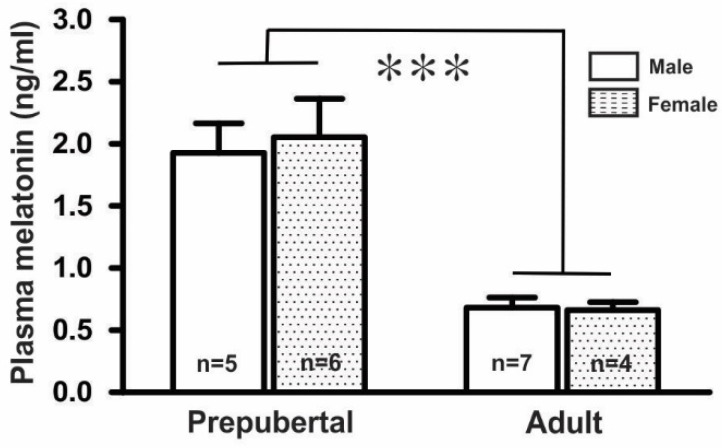
Mean plasma melatonin levels in GnRH-GFP mice (*n* = 4 to 7 in each group, *** *p* < 0.001; unpaired *t*-test). *n*, the number of mice.

Measurement of plasma melatonin levels in prepubertal and adult mice in both sexes showed that the plasma melatonin levels (1.99 ± 0.25 ng/mL, *n* = 11) in prepubertal mice were threefold higher than those (0.67 ± 0.06 ng/mL, *n* = 11) in adult mice. However, there were no significant differences between the male and female groups in either the prepubertal (males, 1.93 ± 0.24 ng/mL, *n* = 5; females, 2.05 ± 0.31 ng/mL, *n* = 6) or adult mice (males, 0.68 ± 0.08 ng/mL, *n* = 7; females, 0.66 ± 0.07 ng/mL, *n* = 4), as shown in Figure 1.

### 2.2. Effect of Melatonin on the Spontaneous Firing Activities of GnRH Neurons

**Figure 2:**
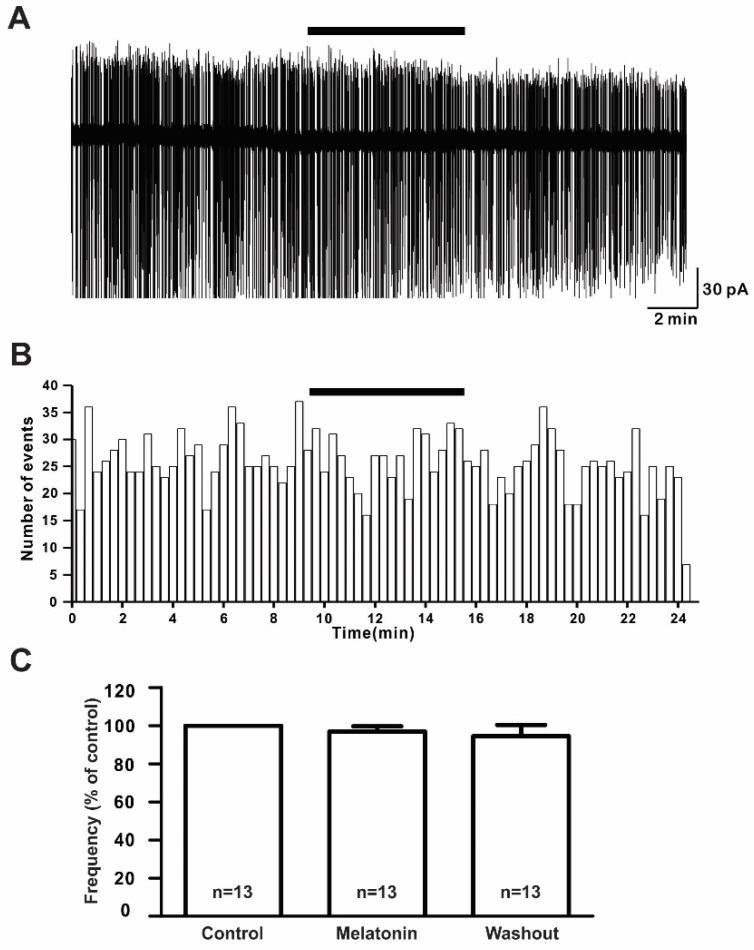
Melatonin did not change the firing activities of gonadotropin-releasing hormone (GnRH) neurons. (**A**) A representative trace of action currents from a GnRH neuron after 20 µM melatonin application in the cell-attached mode. The black bar represents the presence of melatonin. (**B**) Spike frequency histogram (bin size 20 s) of the current trace in Figure 2A. (**C**) Bar graph showing the firing frequency percentages in the presence and washout of melatonin (*p* > 0.05; one-way ANOVA). *n*, the number of neurons tested.

To assess the effect of melatonin on the firing activities of GnRH neurons in prepubertal mice, we applied 20 μM melatonin to 13 GnRH neurons for 5 to 7 min. There were no noticeable changes in the spontaneous activities or relative mean firing frequency after melatonin application (% of control ± standard error of the mean (SEM): 20 μM melatonin: 97.0 ± 3.0%, washout: 95.0 ± 6.0%; Figure 2A,B) (*n* = 13, *p* > 0.05; one-way ANOVA, Figure 2C).

### 2.3. Effect of Melatonin on the Glutamate Receptor Agonist-Mediated Responses in GnRH Neurons

**Figure 3:**
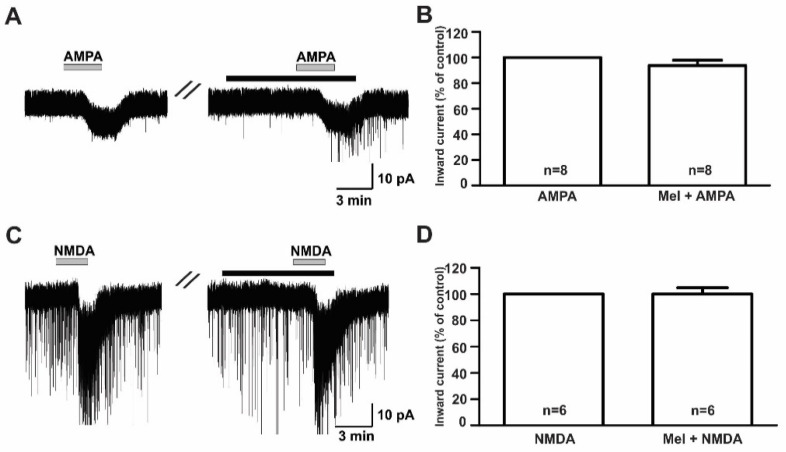
Effects of melatonin on α-amino-3-hydroxy-5-methyl-4-isoxazolepropionic acid (AMPA)- and N-methyl-D-aspartic acid (NMDA)-induced responses. (**A**,**C**) Representative traces showing inward currents induced by AMPA (10 µM) and NMDA (30 µM) in the absence and the presence of 10 µM melatonin. The black bars represent the duration of the melatonin application. (**B**,**D**) Bar graphs showing the mean relative percentage of inward currents induced by AMPA and NMDA in the absence and the presence of 10 µM melatonin (*p* > 0.05; paired *t*-test). *n*, the number of neurons tested; Mel, melatonin.

To investigate the modulatory effect of melatonin on the excitatory neurotransmission of GnRH neurons in prepubertal mice, we investigated the effects of melatonin on excitatory neurotransmitters using ionotropic glutamate receptor agonists such as AMPA, NMDA, and kainate. Under the whole-cell voltage clamp, the bath application of AMPA (10 µM) induced inward currents with a mean amplitude of −8.59 ± 0.72 pA (*n* = 8). Pretreatment with melatonin (10 µM) for 5 to 7 minutes did not change the AMPA-mediated responses (Figure 3A, AMPA, 8.64 ± 0.82 pA; melatonin + AMPA, −7.61 ± 0.88 pA). The mean relative percentage of inward current induced by AMPA in the presence of melatonin compared to AMPA alone was 89 ± 6.0% (*n* = 8, *p* > 0.05; paired *t*-test, Figure 3B). Similar to AMPA, the 30 µM NMDA-induced inward currents were not altered by the presence of melatonin (Figure 3C). The mean relative percentage of inward current induced by NMDA in the presence of melatonin compared to NMDA alone was 100 ± 5.0% (*n* = 6, *p* > 0.05; paired *t*-test, Figure 3D).

**Figure 4:**
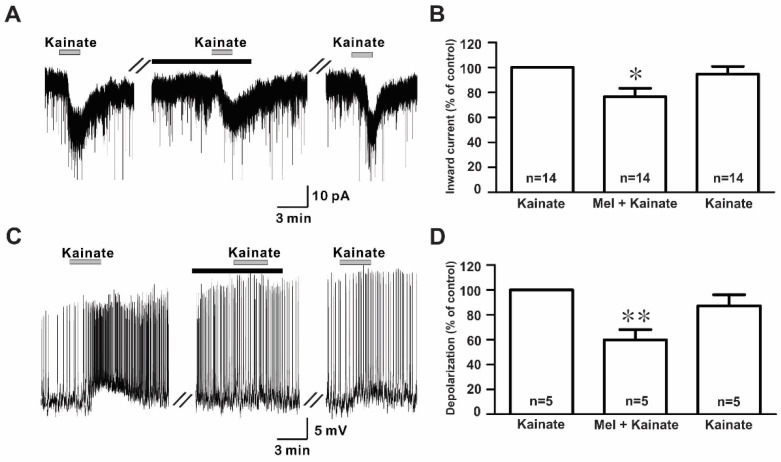
Effects of melatonin on kainate-induced responses. (**A**) A representative trace showing inward currents induced by kainate (10 µM) in the absence and the presence of 10 µM melatonin. The black bar represents the duration of the melatonin application. (**B**) Bar graph showing the mean relative percentage of inward currents induced by kainate in the presence of 10 µM melatonin and after washout of melatonin compared to kainate alone (* *p* < 0.05; one-way ANOVA). (**C**) A membrane potential trace showing membrane depolarization induced by kainate (10 µM) in the absence and the presence of 10 µM melatonin in the gramicidin-perforated current clamp mode. The black bar represents the duration of the melatonin application. (**D**) Bar graph showing the relative percentage of mean depolarizations induced by kainate in the presence of 10 µM melatonin and after melatonin washout compared to kainate alone (* *p* < 0.05, ** *p* < 0.01; one-way ANOVA). *n*, the number of neurons tested; Mel, melatonin.

The bath application of kainate (10 µM) evoked inward currents with a mean amplitude of −17.9 ± 1.60 pA (*n* = 14) in the whole-cell voltage clamp mode (Figure 4A). However, the co-application of melatonin reduced the kainate-induced response, which was recovered after a melatonin washout. The mean relative percentages of inward currents induced by kainate in the presence of melatonin and melatonin washout compared to kainate alone were 76.4 ± 6.7% and 98.1 ± 8.7%, respectively (*n* = 14, *p* < 0.05; one-way ANOVA, Figure 4B). Similarly, in the gramicidin-perforated current clamp mode, melatonin reduced kainate-evoked depolarizations (kainate, 4.98 ± 0.69 mV; melatonin + kainate, 2.79 ± 0.22 mV; washout, 4.20 ± 0.46 mV; *n* = 5, *p* < 0.05; one-way ANOVA, Figure 4C). The mean relative percentages of membrane depolarizations induced by kainate in the presence of melatonin and melatonin washout compared to kainate alone were 60.0 ± 8.2% and 87.0 ± 8.8%, respectively (*n* = 5, *p* < 0.05; one-way ANOVA, Figure 4D). Under whole-cell voltage clamp mode, no significant difference was seen in the inhibition of kainate-induced responses by melatonin between males (77.4 ± 9.9%, *n* = 8) and females (75.2 ± 9.7%, *n* = 6) compared to kainate alone. Therefore, the data were collectively presented.

**Figure 5:**
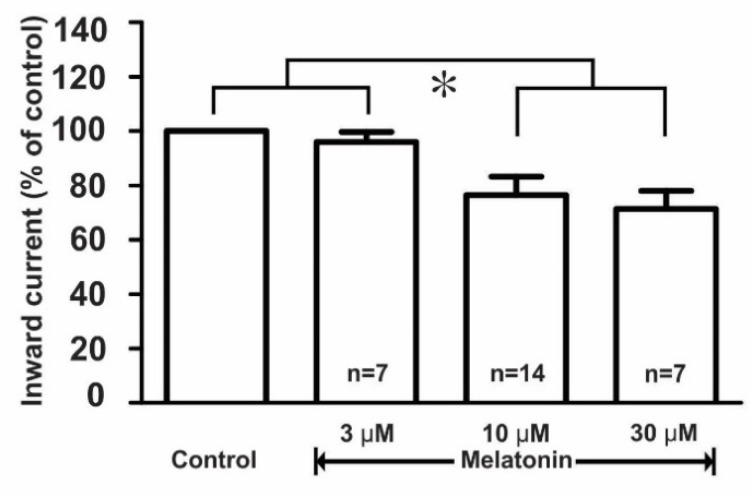
Histogram showing the mean relative percentages of inward currents induced by kainate (10 µM) in the absence and the presence of various concentrations (3, 10, and 30 µM) of melatonin. (* *p* < 0.05; one-way ANOVA post-hoc Scheffe test). *n*, the number of neurons tested.

Further, the effect of melatonin on the kainate receptor-mediated response was dose-dependent where the applications of 3, 10, and 30 μM concentrations of melatonin evoked a concentration-dependent inhibition of kainate action on GnRH neurons. The mean relative percentages of inward current induced by kainate in the presence of 3, 10, and 30 μM melatonin compared to those of kainate alone were 96.0 ± 4.0% (*n* = 7), 76.4 ± 6.7% (*n* = 14), and 71.0 ± 6.7% (*n* = 7), respectively (*p* < 0.05; one-way ANOVA post-hoc Scheffe test, Figure 5). 

### 2.4. Melatonin Directly Acted on Post-Synaptic GnRH Neurons

**Figure 6:**
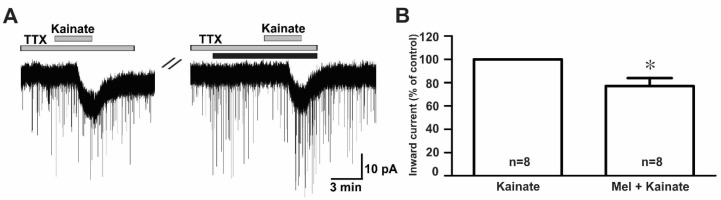
Direct melatonin action on the ionotropic glutamate receptor-mediated effect on GnRH neurons. (**A**) A representative current trace showing inhibition of the kainate (10 µM)-induced response by 10 µM melatonin in the presence of tetrodotoxin (TTX) (0.5 µM), a voltage-sensitive sodium channel blocker. The black bar represents the duration of the melatonin application. (**B**) Bar graph showing the mean relative percentage of inward currents induced by kainate in the presence of melatonin + TTX compared to kainate in the presence of TTX (* *p* < 0.05; paired *t*-test). *n*, the number of neurons tested.

To examine whether melatonin directly modulated the effect of ionotropic glutamate receptor agonists on GnRH neurons, we investigated the effect of melatonin in the presence of tetrodotoxin (TTX), a voltage-gated sodium channel blocker. We found that melatonin suppressed the kainate receptor-mediated response in the presence of TTX (TTX + kainate, −14.1 ± 1.26 pA; TTX + melatonin + kainate, −10.9 ± 1.56 pA, *n* = 8, *p* < 0.05; paired *t*-test, Figure 6A). The mean relative percentage of inward currents induced by kainate in the presence of TTX and melatonin compared to kainate with TTX was 76.0 ± 6.0% (*n* = 8, *p* < 0.05; paired *t*-test, Figure 6B).

### 2.5. Melatonin Effect on the Kainate Response Was Independent of Its Own Receptors but PTX Sensitive

**Figure 7:**
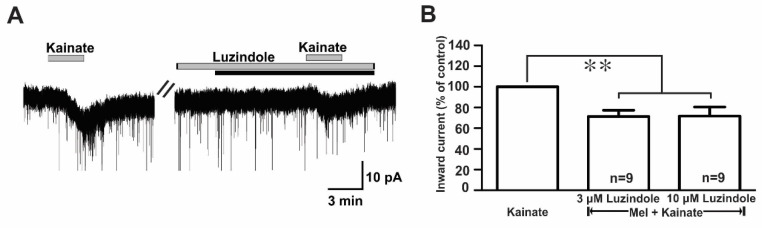
Luzindole, a non-selective melatonin receptor antagonist, did not change the melatonin effects on kainate-induced responses. (**A**) A representative trace showing no effect of 10 µM luzindole on the melatonin (10 µM) inhibition of 10 µM kainate-induced responses. The black bar represents the duration of the melatonin application. (**B**) Bar graph showing the mean relative percentages of inward currents induced by kainate in the presence of melatonin + luzindole compared to kainate in the presence of luzindole alone (** *p* < 0.01; paired *t*-test). *n*, the number of neurons tested; Mel, melatonin.

Melatonin exerts its influence by binding to two specific G protein-coupled receptors (GPCRs), namely, MT_1_ and MT_2_ [10]. To assess which melatonin receptors were involved in the suppression of kainate response, we used luzindole, a melatonin MT_1_ receptor antagonist, or an MT_2_ antagonist. In the presence of luzindole (3 µM and 10 µM), the melatonin-induced suppression of kainate receptor-mediated responses was maintained (Figure 7A). The mean relative percentages of inward currents induced by kainate in the presence of luzindole and melatonin compared to kainate with luzindole were 71.0 ± 6.0% (3 µM luzindole, *n* = 9) and 71.0 ± 8.0% (10 µM luzindole, *n* = 9), respectively (*p* < 0.05; paired *t*-test, Figure 7B).

**Figure 8:**
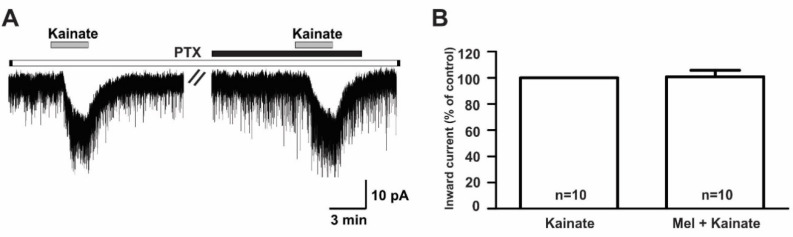
The melatonin effect on kainate-induced responses was blocked by the presence of pertussis toxin, a G-protein-coupled receptor blocker. (**A**) A representative trace showing the blockade of 10 µM melatonin action on the kainate (10 µM)-induced response in GnRH neurons in brain slices incubated with pertussis toxin for more than 8 h. The black bar represents the duration of melatonin application. (**B**) Bar graph showing the mean relative percentage of inward current induced by kainate in the presence of melatonin compared to kainate alone, following pre-incubation with pertussis toxin (*p* > 0.05; paired *t*-test). *n*, the number of neurons tested; Mel, melatonin.

In contrast, the suppressive effect of melatonin on the kainate receptor-mediated responses was completely blocked when recordings were made from slices pre-incubated (>8 h) with 100 ng/mL pertussis toxin (PTX), a G-protein-coupled receptor blocker (kainate, −18.3 ± 2.63 pA; melatonin + kainate, −18.1 ± 2.67 pA; *n* = 10, *p* > 0.05; paired *t*-test, Figure 8A). The mean relative percentage of inward current induced by kainate in the presence of melatonin compared to that of kainate alone was 100 ± 5.0% (*n* = 10, *p* > 0.05; paired *t*-test, Figure 8B).

## 3. Discussion

The results of the present study showed that melatonin acted as a negative regulator of hypothalamic GnRH neurons in prepubertal mice. Although melatonin alone did not change the electrical activities of GnRH neurons, the excitatory response mediated by the kainate receptor was suppressed by the effect of melatonin on the GnRH neurons. In addition, the melatonin inhibition of kainate-mediated responses was action potential-independent, suggesting that melatonin acted directly on post-synaptic GnRH neurons. Furthermore, the melatonin effect on kainate receptors was a melatonin receptor-independent PTX-sensitive G-protein-coupled action.

Neurotransmitters and neuropeptides found in the hypothalamus are known as GnRH secretion-activating and -inhibiting factors [25]. Glutamate, the major excitatory neurotransmitter, plays a pivotal role in the regulation of the reproductive axis via acting on the GnRH neuronal network [27]. In addition, GnRH neurons carry glutamate receptors that control their excitability, and hence GnRH secretion from the median eminence [2]. Furthermore, the activation of ionotropic glutamate receptors regulates both the pulse and the surge modes of GnRH secretion [28,29] involved in the critical processes of puberty, pulsatile hormone release, and sexual behavior [30].

Premature activation of the normal HPG axis results in the pulsatile secretion of GnRH and subsequent activation of the gonads, causing central precocious puberty (CPP) [31]. Excitatory amino acids have stimulatory effects on the reproductive axis, particularly at the time of puberty, and their over-activation is linked to CPP [4]. At the prepubertal stage, elevated melatonin levels maintain the hypothalamus in a quiescent state and influence the maturation of the HPG axis [32]. Human studies indicated that during the pre-pubertal phase, serum melatonin levels peaked and decreased progressively, and in a significant way, were proportional to sexual maturation, which led to the onset of pubertal changes [33]. Similarly, high plasma melatonin levels are associated with prepubertal and delayed pubertal conditions, whereas a low level of melatonin is associated with the post-pubertal state or CPP [34].

In this study, we found that the plasma melatonin levels in prepubertal mice were about three times higher than that of the adults. The average plasma melatonin level of adult mice was 0.67 ng/mL, which was slightly higher than that in previous reports [35]. This discrepancy could be explained by the sensitivities of the methods used, the age of the mice, and time of the blood collections. Melatonin levels in laboratory mice vary depending upon the strains and the time of measurement [36,37]. Regardless of whether C57BL/6 mice are considered a melatonin-deficient or non-deficient strain of mice, the anti-gonadal action of exogenous melatonin has been reported in a melatonin-deficient strain of mice [38]. Our data showed that the GnRH-GFP mice with C57Bl6/DBA genetic background mice used in these experiments had measurable plasma melatonin levels. This study also demonstrated that the melatonin concentration in pre-pubertal mice were higher than those in adult mice. Similar age-dependent changes in melatonin levels have been reported in rodents and humans [39,40,41].

In several neuronal populations, melatonin has been found to control neuronal excitabilities, synaptic events, and neurotransmitter release in concentrations ranging from nanomolar to millimolar [42,43,44,45,46,47,48,49,50]. For example, the γ-aminobutyric acid (GABA) response was inhibited by melatonin in nanomolar to micromolar ranges in brain slices of rat trigeminal ganglion neurons [45] and GnRH neurons [46]. In addition, a study reported pharmacological concentrations of melatonin from 1 µM to 100 µM in neural stem cells [51]. In this study, when 10 µM melatonin was co-applied with glutamate receptor agonists, the NMDA and AMPA receptor-mediated actions remained unaffected. In contrast, the application of 10 and 30 µM melatonin significantly reduced kainate-mediated responses, with no effect at 3 μM.

Excitatory neurotransmission is regulated by both pre- and post-synaptically expressed kainate receptors, which control neuronal network activity by regulating neurotransmitter release [52]. In this study, in the presence of TTX (a voltage-sensitive Na^+^ channel blocker), melatonin still exerted a suppressive effect on the kainate-mediated response, suggesting direct post-synaptic melatonin action on the kainate receptors on GnRH neurons. A similar result was reported for the post-synaptic action of melatonin on the inhibition of NMDA receptor-mediated current in substantia gelatinosa (SG) neurons [50].

To examine the receptor mechanism of melatonin action, we applied luzindole, a non-specific melatonin receptor antagonist that blocks both subtypes of melatonin receptors at higher concentrations (>1 µM) [46]. Interestingly, in this study, we observed that luzindole did not block the melatonin inhibition of kainate-mediated responses, suggesting that the melatonin suppression of kainate currents was independent of the melatonin receptors. Melatonin actions independent of melatonin receptors have been reported in the brain [50,53,54]. For example, melatonin enhanced glutamate currents at picomolar and nanomolar concentrations via MT_1_ receptors but suppressed the glutamate currents at micromolar concentrations by binding to the allosteric sites on the AMPA receptors [53]. Additionally, luzindole did not diminish the suppressive effect of melatonin on NMDA receptor-mediated current in SG neurons [50].

Since melatonin action on the kainate receptor-mediated responses was melatonin receptor-independent, we hypothesized that melatonin could act via different G-protein-coupled receptors (GPCRs). Ionotropic glutamate receptors are regulated by several GPCR pathways that involve a variety of intercellular signaling molecules and regulatory proteins that vary from one cell type to another [55]. The coupling of kainate receptors to G-proteins has been observed in the rat hippocampus [56] and the goldfish brain [57]. Similarly, G-proteins that are linked to kainate receptors are PTX-sensitive G-proteins [56,57]. In addition, melatonin regulates cell function through intracellular second messengers, such as cyclic adenosine monophosphate (cAMP), Ca^2+^, cyclic guanosine monophosphate (cGMP), diacylglycerol, protein kinase C (PKC), and arachidonic acid, and its effect on second messengers is mediated by PTX-sensitive G-protein signaling [58]. Indeed, we observed the complete abolishment of the melatonin effect on kainate receptor-mediated responses in GnRH neurons from brain slices pre-incubated with the GPCR inhibitor, pertussis toxin. This indicates the involvement of G-proteins in the melatonin inhibition of the kainate receptor-mediated response in GnRH neurons.

Our single-cell electrophysiological studies suggest that melatonin may play a role in the puberty process via regulating glutamatergic input in pre-pubertal GnRH neurons. However, further studies are required to determine the specific G-protein and downstream signaling pathways in regulating glutamatergic input by melatonin. Moreover, our study also demonstrated that the melatonin levels in adult mice were lower than those in pre-pubertal mice. This suggests a possible role of melatonin in the regulation of GnRH neurons in puberty, which warrants additional investigations. Taken together, our results demonstrated that plasma melatonin levels decreased with age and that melatonin exerted inhibitory action on pre-pubertal hypothalamic GnRH neurons by suppressing kainate receptor-mediated responses through G-proteins, providing evidence that melatonin could directly regulate the HPG axis at the hypothalamic GnRH neuron level.

## 4. Materials and Methods

### 4.1. Animals

Prepubertal postnatal day 10 to 25 (PND10 to PND25) and adult (PND80) male and female GnRH-GFP mice with C57Bl6/DBA genetic background mice [59], housed under 12 h light–12 h dark cycle (lights on at 07:00 a.m.) with ad libitum access to food and water were used for the experiments. All animal care conditions and experiments were approved by the Institutional Animal Care and Use Committee of Jeonbuk National University, CBNU 2018-071 (2018-09-04), CBNU 2019-089 (2019-11-14), and CBNU 2019-054 (2019-07-30).

### 4.2. Enzyme-Linked Immunosorbent Assay (ELISA)

To verify the presence of melatonin in GnRH-GFP mice, we measured plasma melatonin levels in prepubertal and adult mice of both sexes. The melatonin levels were measured using an ELISA kit (ab213978, Lot: GR3271599-3, Abcam, Cambridge, MA, USA). For the melatonin assay, the blood was collected by retro-orbital bleeds from prepubertal (PND15) and adult (PND80) GnRH-GFP mice at 5:00 p.m. UTC+09:00 (Universal Time Coordinated). The blood sample volumes collected per animal, especially from PND15 mice, were limited. Approximately 0.2 mL and 0.25 mL of blood could be collected from each PND15 and PND80 GnRH-GFP mouse, respectively. Since the ELISA measurements in this study required 0.2 mL of blood per animal, we had one sample from each mouse. The collected blood was transferred to microcentrifuge tubes (Sarstedt, Nümbrecht, Germany). The blood samples were centrifuged at 1000 × *g* for 10 min at 4 °C to separate the plasma. An equal volume of cold ethyl acetate (100 μL) was added to each plasma sample (100 μL), and gently mixed. The samples were incubated on ice for 3 minutes to allow the layers to separate. All samples were mixed again by vortexing, incubated on ice for 2 minutes, and centrifuged at 1000 × *g* for 10 min at 4 °C. The organic layer was removed to a new tube, and the samples were vacuum centrifuged completely. The dried extract was resuspended in 100 μL of 1× kit stabilizer and the assay was conducted according to the manufacturer’s instructions. The optical density of the plate was read at 450 nm (Molecular Devices, San Jose, CA, USA).

The sensitivity of the melatonin kit and the range are 162 pg/mL and 0.08–50 ng/mL, respectively, with an intra-assay variation of 4.31%, according to the manufacturer. Due to the blood sample volume limitation, we were not able to run duplicates. However, all standard samples were duplicated to ensure the curve fit. The intra-assay CV% value of the standards samples was less than 4.97%.

### 4.3. Brain Slice Preparation

Coronal brain slices containing the preoptic area were prepared for patch clamp recording, as described previously [60]. The mice were decapitated and their brains were quickly excised and immersed in ice-cold artificial cerebrospinal fluid (ACSF) with the following composition (in mM): 126 NaCl, 2.5 KCl, 2.4 CaCl_2_, 1.2 MgCl_2_, 11 D-glucose, 1.4 NaH_2_PO_4_, and 25 NaHCO_3_ (pH 7.3–7.4 maintained by bubbling with 95% O_2_ and 5% CO_2_). Coronal slices (230 μm in thickness) containing the preoptic hypothalamic area were cut using a vibratome (VT1200S; Leica biosystem, Wetzlar, Germany) in ice-cold ACSF. The slices were allowed to recover in oxygenated ACSF at room temperature for at least 1 h before electrophysiological recording.

### 4.4. Electrophysiology

Each brain slice was transferred to the recording chamber, entirely submerged, and continuously superfused with oxygenated ACSF at a flow rate of 4 to 5 mL/min. The coronal slices were viewed with an upright microscope (BX51W1; Olympus, Tokyo, Japan) and the images were displayed on a video monitor. Fluorescent GnRH neurons were identified under X10 and X40 objective magnifications via brief fluorescence illumination and patched under Nomarski differential interference contrast optics. The patch pipettes were made from thin-walled borosilicate glass capillary tubing (PG52151-4; WPI, Sarasota, FL, USA) on a Flaming/Brown puller (P-97; Sutter Instruments Co., Novato, CA USA). The pipette solution was composed of (in mM) 140 KCl, 1 CaCl_2_, 1 MgCl_2_, 10 4-(2-Hydroxyethyl)piperazine-1-ethanesulfonic acid (HEPES), 4 Mg-ATP, and 10 ethylene glycol-bis (2-aminoethyl ether)-N,N,N’,N’-tetraacetic acid (EGTA) (pH 7.3 adjusted with KOH), and filtered through a disposable 0.22 µM filter. The tip resistance of the electrode ranged from 4 to 6 MΩ. The electrode potentials were nullified before the gigaseal was achieved, and the neurons were voltage-clamped at −60 mV for the whole-cell patch clamp using an Axopatch 200B (Molecular Devices, San Jose, CA, USA). The cell membrane patch was ruptured under negative pressure by a short suction pulse for whole-cell patch clamp recordings. For perforated patch recording, gramicidin (Sigma-Aldrich, St. Louis, MO, USA) was first dissolved in dimethyl sulfoxide (DMSO; Sigma-Aldrich) to a concentration of 2.5 to 5 mg/mL and diluted to a final concentration of 2.5 to 5 µg/mL in the pipette solution just before use and sonicated for 10 min. In the initial experiment, the access resistance was monitored, and the experiments were initiated when the resistance stabilized between 50 and 90 MΩ. Typically, it took 20 to 35 min after gigaseal formation and always corresponded to the resting membrane potential of the cell, reaching a stable level below −45 mV. A sudden overshooting of action potentials above 0 mV suggested the spontaneous rupture of the membrane. To evoke spontaneous action current firings, we performed cell-attached recordings in voltage clamp mode at a holding potential of 0 mV. Signals (voltage and current) were amplified with an Axopatch 200B and filtered at 1 kHz with a Bessel filter before digitizing at a rate of 1 kHz. The membrane potential and membrane current changes were sampled using a Digidata 1440A interface (Molecular Devices, San Jose, CA, USA) connected to a desktop PC. Acquisition and the subsequent analysis of the acquired data were performed using Axon pClamp 10.6 software (Molecular Devices, San Jose, CA, USA). Traces were plotted using Origin 8 software (OriginLab Corp, Northampton, MA, USA). All recordings were made at room temperature.

Effect of melatonin on the firing activities of GnRH neurons was assessed by perfusion of 20 μM melatonin for 5 to 7 min. Excitatory neurotransmitter-mediated responses were evoked by perfusion of ionotropic glutamate receptor agonists such as AMPA (10 µM), NMDA (30 µM), and kainate (10 µM). NMDA-mediated currents were recorded in the presence of Mg^2+^-free ACSF solution and 1 µM glycine, as Mg^2+^ may induce a voltage-dependent block of the NMDA response [61] and a glycine-induced intense NMDA response with reduced NMDA receptor desensitization [62]. To decide the postsynaptic effect of melatonin, we perfused 0.5 µM tetrodotoxin (TTX), a voltage-gated sodium channel blocker, during that experiment. For the melatonin receptor-based experiment, a non-selective melatonin receptor antagonist, luzindole (3, 10 µM), was perfused 5 min prior to melatonin application.

### 4.5. Chemicals

Melatonin, luzindole, NMDA, kainic acid, and glycine were purchased from Sigma-Aldrich. AMPA, tetrodotoxin citrate (TTX), and pertussis toxin (PTX) were purchased from Tocris Bioscience (Avonmouth, Bristol, United Kingdom). Melatonin and luzindole were dissolved in DMSO and the other chemicals were dissolved in distilled water. The stocks were diluted (usually 1000-fold) in ACSF to a desired final concentration before bath application. The stock concentration of melatonin was 50 mM.

### 4.6. Statistical Analysis

The neuronal firing was analyzed using Mini-Analysis software (ver. 6.0.7; Synaptosoft Inc., Decatur, GA, USA). An equivalent period (5 min) was set for the analysis of firing in the control, melatonin application, and washout. The relative percentage was calculated by dividing the target response by its control response and multiplying by 100. For statistical analysis, Student’s *t*-test and the one-way ANOVA post-hoc Scheffe test were used to compare the means of two and more than two experimental groups, respectively. All values are expressed as the mean ± SEM. A *p*-value of <0.05 was considered statistically significant. Statistical analyses were performed using Origin 8 software (OriginLab Corp, Northampton, MA, USA).
